# Metabolic obesity phenotypes and chronic kidney disease: a cross-sectional study from the RaNCD cohort study

**DOI:** 10.1186/s12882-022-02858-9

**Published:** 2022-07-01

**Authors:** Samira Arbabi Jam, Behrooz Moloudpour, Farid Najafi, Mitra Darbandi, Yahya Pasdar

**Affiliations:** 1 Student Research Committee, Kermanshah University of Medical Sciences, Kermanshah, Iran; 2 Student Research Committee, Tabriz University of Medical Sciences, Tabriz, Iran; 3 Research Center for Environmental Determinants of Health (RCEDH), Health Institute, Kermanshah University of Medical Sciences, Kermanshah, Iran; 4 Cardiovascular Research Center, Kermanshah University of Medical Sciences, Kermanshah, Iran

**Keywords:** Metabolically healthy, Obesity phenotypes, Kidney function, PERSIAN

## Abstract

**Background:**

Investigating the effect of metabolic disorders on chronic kidney disease (CKD) in the presence or the absence of obesity is of great importance. This study aimed to examine the independent and joint relationships of obesity and metabolic syndrome (MetS) with CKD.

**Methods:**

The present study was performed on 9,762 participants from the baseline phase of the Ravansar non- communicable diseases (RaNCD) study. Thereafter, the CKD was estimated by glomerular filtration rate (eGFR) using the Modification of Diet in Renal Disease (MDRD) equation. All the included participants were categorized into the following four phenotypes: metabolically healthy non-overweight/obesity (MHNO), metabolically unhealthy non-overweight/obesity (MUNO), metabolically healthy overweight/obesity (MHO), and metabolically unhealthy overweight/obesity (MUO). Finally, Logistic regression analysis was used to estimate the odds ratio (ORs).

**Results:**

The mean age of the included participants was 47.33 ± 8.27 years old, %48.16 (4,701) of whom were men. As well, 1,058(10.84%) participants had CKD (eGFR less than 60 ml/min/1.73m^2^). The overweight/obesity was not significantly associated with odds of CKD. The odds of CKD in male subjects with MetS was 1.48 times higher than non-MetS ones (95% CI: 1.10, 2.01). After adjusting the confounders, the odds of CKD were 1.54 times (95% CI: 1.12, 2.11) higher in the MUNO and 2.22 times (95% CI: 1.44, 3.41) higher in the MUO compared to MHNO phenotype in male subjects. The odds of CKD in the MUNO and MUO was 1.31 times (95% CI: 1.10, 1.60) and 1.23 times (95% CI: 1.01, 1.54) higher than MHNO phenotype in female subjects, respectively.

**Conclusion:**

The odds of CKD were higher in MUNO and MUO phenotypes. Therefore, lifestyle modification is recommended to control normal weight and healthy metabolism.

## Introduction

The term chronic kidney disease (CKD) is used for a wide range of kidney diseases characterized by the gradual loss of functional nephrons [1]. The Global Burden of Disease (GBD) study in 2017 has reported that approximately 700 million people have CKD worldwide [2]. In a population-based study conducted on 30,041 Iranian (2021), the prevalence of CKD stage III + was estimated to be 7.1 and 5.5 based on Diet in Renal Disease (MDRD) and CKD Epidemiology Collaboration (CKD-EPI), respectively [3]. It is noteworthy that the CKD may eventually progress to end-stage renal disease (ESRD), in which patients survive if kidney substitutes such as dialysis or transplants are applied. Moreover, CKD can play important roles in morbidity and mortality in populations [4, 5]. The CKD-EPI and MDRD are the two widely-used, accurate methods for estimating GFR, which are applied in large and diverse populations [6, 7]. Therefore, the estimation of glomerular filtration rate (eGFR) based on endogenous filtration markers like serum creatinine, is often applied as a measure of general kidney function in both clinical and population-based studies [8–10].

Studies have previously reported different risk factors for CKD, including age, hypertension, low HDL serum, type 2 diabetes mellitus (T2DM), and central obesity (waist and hip circumferences) [11–13]. Among the above-mentioned risk factors, obesity is the most important factor. Accordingly, obesity is the cause of many non- communicable diseases (NCDs), which is also effective on the development of kidney disease [14, 15]. Although the mechanisms by which obesity causes the occurrence and exacerbation of CKD are still unknown, aligning obesity with metabolic risk factors and CVDs may partly help to identify the related mechanisms, and this requires performing extensive studies in diverse populations. Therefore, a direct cause of kidney disease should be clarified; either it is obesity or metabolic disorders caused by obesity [16, 17]. Scientific report suggested that obesity, due to its anti-inflammatory and oxidative stress effects, can lead to the development of dyslipidaemia, insulin resistance (IR), and other metabolic disorders, which in general metabolic unhealthy obesity (MUO) is considered [18, 19]. A retrospective cohort study performed on Japanese population (2015), indicated that the metabolically unhealthy obese (MUO) phenotype, unlike the metabolically healthy obese (MHO) phenotype, is associated with the risk of CKD [14]. Nevertheless, a study conducted on 42,128 adults in Pennsylvania found that metabolically healthy obesity (MHO) is associated with the increased risk of kidney disease, regardless of either the presence or the absence of metabolic unhealthy [20]. These contradictions may possibly be due to differences in populations, ethnicities, or sample sizes, which require further investigations. Therefore, the current study aimed to assess the association between metabolic obesity phenotypes and CKD among Iranian Kurdish adults.

## Methods

### Study design and population

This cross-sectional study was conducted using the baseline phase data obtained from the Ravansar non- communicable diseases (RaNCD) cohort study in 2021. Ravansar is a city with 50,000 populations, located in Kermanshah province, western Iran. Of note, the RaNCD cohort study is a 15-year prospective epidemiological study, the baseline phase of which was started in 2014. The sample size of the study was estimated as 10,047 people, which was established to study a prospective national group (PERSIAN)^1^ in people aged between 35 and 65 years old in Ravansar. A detailed description of the design of RaNCD study has been published previously [21].

**Fig. 1:**
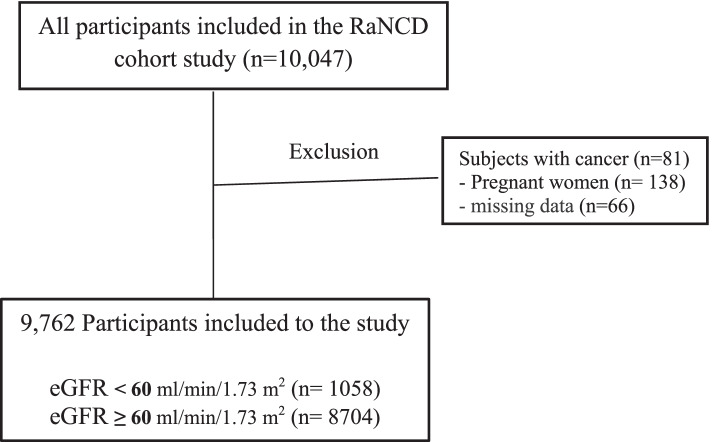
Flow chart of study

Of the total RaNCD participants, 285 cases were excluded from the present study due to the following reasons: participants with cancer (*n* = 81), pregnant women (*n* = 138), and subjects with incomplete information (*n* = 66). Finally, 9762 participants were included in the present study (Fig. 1).

### Data collection and measurements

Demographic information of the participants was collected by the trained experts of RaNCD cohort centre using a digital questionnaire. Moreover, blood pressure, height, weight, and BMI were measured in terms of the cohort profile [21].

Socio-economic status (SES): The socio-economic status (SES) was created by 18 items using principal component analysis (PCA) method. Next, the SES was categorized in three groups ordered from the lowest to the highest one.

Smoking and alcohol consumption: Current smokers were people who reported they had smoked at least 100 cigarettes. Alcohol consumption is defined as drinking approximately 200 ml of beer OR 45 ml of alcohol, once per week for at least six months.

Physical activity: To assess physical activity, at first, a standard 22-item questionnaire of Ravansar cohort was categorized into the following three levels: low (24–36.5 MET/hour per day), medium (36.6–44.4 MET/hour per day), and high (≥ 44.5 MET/hour per day) according to Met/hour per day.

Nutritional information: The nutritional information and energy intake level were collected using Food Frequency Questionnaire (FFQ) with 118 items.

Biochemical measurements: After 12 h of fasting, biochemical markers, including creatinine (Cr), blood urea nitrogen (BUN), high-density lipoprotein Cholesterol (HDL-C), low-density lipoprotein Cholesterol (LDL-C), triglyceride (TG), Total cholesterol (T-C), fasting blood sugar (FBS), γ-glutamyltransferase (GGT), alanine aminotransferase (ALT), and aspartate aminotransferase (AST) were measured.

Muscle strength: Muscle strength (Handgrip strength) was measured using a digital dynamometer (Model: Saehan, SH5003, Saehan Co, South Korea). Anthropometric indices, including weight, BMI, body fat mass (BFM), Fat free mass (FFM), and waist-to-hip ratio (WHR) were measured using bioelectric impedance InBody 770 model (Inbody Company, Seoul, Korea).

### Obesity phenotypes

At this stage, general obesity was measured by BMI (kg/m^2^) and then classified into normal weight (BMI: 18.5–24.9), overweight (BMI: 25–29.9), and obesity (BMI: ≥ 30). Metabolic health was defined in terms of the International Diabetes Federation (IDF) criteria for MetS [22]. Subsequently, four phenotypes (BMI-MetS categories) were categorized based on BMI (normal weight, overweight/obese) and MetS (yes/no) as follows: (1) MHNO; MHO; MUNO; MUO.

## Definitions

In the present study, the definitions were provided in terms of the RaNCD cohort study protocol, so that the participants with a history of hospitalization and/or treatment for one or more types of heart diseases such as stroke, Myocardial Infarction (MI), and coronary artery disease, and/or those who were consuming medications for these conditions, were considered as CVDs [23]. Hypertension was defined as SBP ≥ 140 mmHg and/or DBP ≥ 90 mmHg and/or those who were using antihypertensive drugs [24]. Dyslipidaemia was defined as a disorder in lipid profile and/or a history of consuming medication for lipid disorders [25]. In addition, Type 2 diabetes mellitus (T2DM) was defined as Fasting Blood Sugar (FBS) >  = 126 mg/dl and/or history of using medication for T2DM treatment [26]. CKD was estimated by eGFR using the Modification of Diet in Renal Disease (MDRD) equation [10] as follows:

$$\begin{array}{c}175\times \mathrm{Serum} {\mathrm{Cr}}^{-1.154}\times {\mathrm{age}}^{-0.203}\mathrm{for male cases}\\ 175 \times \mathrm{Serum }{\mathrm{Cr}}^{-1.154 }\times {\mathrm{age}}^{-0.203}\times 0.742 \mathrm{for female cases}.\end{array}$$

The decreased kidney function was defined as eGFR less than 60 mL/min/1.73m^2^ in terms of the Kidney Disease Improving Global Outcomes criteria for CKD.

### Statistical analysis

Continuous variables were presented as mean ± standard deviation (SD), and categorical variables were presented as frequency (%). To compare demographic and biochemical characteristics among the four groups of phenotype obesity, one-way analysis of variance (ANOVA) was performed for continuous variables, and the Chi square test was done for categorical variables. The demographic, biochemical characteristics, and comorbidity were compared by t-test between the two groups of eGFR (eGFR less than 60 and equal to or more than 60 ml/min/1.73m^2^) for continuous variables. Additionally, the Chi square test was used for categorical variables. Moreover, logistic regression analysis was performed to determine the association between phenotype obesity and CKD. Regression models were also adjusted for potentially confounding variables, including age, SES, physical activity, smoking, alcohol use, and CVD. The estimations were presented with 95% confidence interval and *P* < 0.05. All these analyses were done using STATA software version 14.2 (Stata Corp, College Station, Tex).

## Results

Table 1 presents the baseline demographic, clinical, biochemical, and nutritional characteristics of the included participants according to eGFR groups. A total of 9,762 participants with a mean age of 47.33 ± 8.27 years old were studied in this research. Correspondingly, %48.16 (4701) of the participants were men, and %11.72 (1138) of them were current smokers. The prevalence rates of hypertension and CVD were significantly lower in the participants with eGFR equal to or more than 60 compared to the less than 60 ml/min/1.73 m^2^ (*P* < 0.001). The means of intake levels of vitamin D and B12 were significantly higher in the participants with eGFR equal to or more than 60 compared to less than 60 ml/min/1.73 m^2^. Table 2 presents the baseline demographic, clinical, biochemical, and nutritional characteristics of the participants based on obesity phenotype categories. The prevalence rates of kidney stones (*P* = 0.007) and CVDs (*P* < 0.001) in the MUNO and MUO groups were significantly higher than those of the MHNO and MHO groups. The mean eGFR was found to be significantly different in these four groups, which was higher in the MHNO group compared to the other three groups (*P* < 0.001). Notably, the means of creatinine, BUN, liver enzymes, sodium, energy intake, and percentage of energy intake from protein and lipid in the four groups of obesity phenotypes were statistically significant (*P* < 0.05).

**Table 1:** Baseline characteristics of the study participants according to glomerular filtration rate value in study population (*n* = 9,762)

Characteristic	Glomerular Filtration Rate Mean± SD / n (%)			*P* value *
	**Total**	**GFR < 60** ml/min/1.73 m^2^	**GFR ≥ 60** ml/min/1.73 m^2^	
**Age (year)**	47.33 ± 8.27	52.21 ± 8.90	46.73 ± 7.99	< 0.001
**Sex:**				
**Male**	4701 (48.16)	234 (4.98)	4467 (95.02)	< 0.001
**Female**	9762 (51.84)	824 (16.28)	4237 (83.72)	
**SES:**				
**Low**	3222 (33.02)	626 (59.17)	2596 (29.84)	< 0.001
**Moderate**	3265 (33.46)	280 (26.47)	2985 (34.31)	
**High**	3271 (33.52)	152 (14.37)	3119 (35.85)	
**Physical activity:**				
**Low**	2956 (30.28)	313 (29.58)	2643 (30.37)	< 0.001
**Moderate**	4624 (47.37)	454 (51.51)	4079 (46.86)	
**High**	2182 (22.35)	200 (18.90)	1982 (22.77)	
**Current smoker**	1138 (11.72)	64 (6.11)	1074 (12.39)	< 0.001
**Alcohol use**	478 (4.90)	14 (1.32)	464 (5.33)	< 0.001
**Dyslipidemia**	4332 (44.38)	499 (47.16)	3833 (44.04)	0.053
**Hypertension**	1542 (15.80)	296 (27.98)	1246 (14.32)	< 0.001
**T2DM**	845 (8.66)	136 (12.85)	709 (8.15)	< 0.001
**CVD**	1658 (16.98)	354 (33.46)	1304 (14.98)	< 0.001
**Kidney stone**	1789 (18.33)	189 (17.86)	1600 (13.38)	0.681
**BMI (kg/m**^**2**^**)**	27.49 ± 4.63	27.53 ± 4.75	27.48 ± 4.61	0.707
**WHR**	0.94 ± 0.10	0.94 ± 0.06	0.94 ± 0.06	0.221
**BUN (mg/dl)**	13.62 ± 4.21	14.72 ± 6.10	13.49 ± 3.91	< 0.001
**Cr (mg/dl)**	0.99 ± 0.22	1.22 ± 0.44	0.97 ± 0.15	< 0.001
**AST (mg/dl)**	21.42 ± 9.10	22.01 ± 8.76	21.34 ± 9.10	0.0261
**ALT (mg/dl)**	24.93 ± 14.78	22.07 ± 13.63	25.27 ± 14.88	< 0.001
**GGT (mg/dl)**	24.73 ± 19.87	24.01 ± 17.90	24.81 ± 20.10	0.208
**Energy intake (Kcal/day)**	2651.74 ± 954.00	2204.86 ± 811.88	2706.14 ± 955.75	< 0.001
**Carbohydrate (%E)**	61.38 ± 0.25	61.31 ± 0.20	61.39 ± 0.10	0.129
**Protein (%E)**	13.78 ± 0.11	13.56 ± 0.07	13.81 ± 0.02	0.001
**Lipid (%E)**	26.82 ± 0.20	27.01 ± 0.18	26.79 ± 0.06	0.279
**Sodium intake (g/day)**	4.77 ± 0.02	5.03 ± 0.04	4.85 ± 0.01	< 0.001
**Vitamin D (IU)**	46.40 ± 0.80	40.73 ± 0.99	47.10 ± 0.34	< 0.001
**Vitamin B12 (mg/day)**	7.42 ± 0.06	6.97 ± 0.16	7.48 ± 0.05	0.032

*Abbreviation*: *BMI* Body mass index, *WHR* Waist hip ratio, *SES* Socioeconomic status, *PA* Physical activity, *ALT* Alanine aminotransferase, *AST* Aspartate aminotransferase, *CVD* Cardiovascular disease, *T2DM* Type 2 diabetes mellitus, *BUN* Blood urea nitrogen, *Cr* creatinine, *GFR* glomerular filtration rate, *GGT* gamma-glutamyl transferase

^*^P- value was obtained t-test or Chi square test

**Table 2:** Baseline clinical, anthropometry and biochemical characteristics of the study participants according to phenotype obesity (*n* = 9,762)

Characteristic	Metabolically healthy		Metabolically unhealthy		*P* value *
	**MHNO** (*n* = 4,717)	**MHO** (*n* = 1,302)	**MUNO** (*n* = 2,438)	**MUO** (*n* = 1,305)	
	**Mean ± SD / n (%)**				
**Age (year)**	46.16 ± 8.21	45.70 ± 7.44	49.68 ± 8.41	48.76 ± 7.78	< 0.001
**Sex:**					
**Male**	2584 (54.97)	345 (7.34)	1334 (28.38)	438 (9.32)	< 0.001
**Female**	2133 (42.15)	957 (18.91)	1104 (21.81)	867 (17.13)	
**SES:**					
**Low**	1594 (33.81)	404 (31.03)	786 (32.27)	438 (33.56)	0.012
**Moderate**	1541 (32.68)	455 (34.95)	792 (32.51)	477 (36.55)	
**High**	1580 (33.51)	443 (34.02)	858 (35.22)	390 (29.89)	
**PA:**					
**Low**	1263 (26.78)	398 (30.57)	823 (33.76)	472 (36.17)	< 0.001
**Moderate**	2137 (45.30)	703 (53.99)	1118 (45.86)	666 (51.03)	
**High**	1317 (27.92)	201 (15.44)	497 (20.39)	167 (12.80)	
**Current smoker**	651 (13.87)	65 (5.01)	325 (13.40)	97 (7.48)	< 0.001
**Alcohol use**	249 (5.28)	36 (2.76)	142 (5.82)	51 (3.91)	< 0.001
**Dyslipidemia**	1186 (25.14)	323 (24.81)	1912 (78.42)	911 (69.81)	< 0.001
**T2DM**	84 (1.780)	34 (2.61)	445 (18.25)	282 (21.61)	< 0.001
**CVD**	298 (6.32)	131 (10.06)	755 (30.97)	474 (36.32)	< 0.001
**Hypertension**	263 (5.58)	76 (5.84)	739 (30.31)	464 (35.56)	< 0.001
**Kidney stone**	830 (17.60)	212 (16.28)	485 (19.89)	262 (20.08)	0.007
**BMI (kg/m**^**2**^**)**	24.83 ± 3.15	33.10 ± 3.02	26.45 ± 2.41	33.38 ± 3.27	< 0.001
**WHR**	0.91 ± 0.05	0.99 ± 0.05	0.94 ± 0.05	0.99 ± 0.06	< 0.001
**Muscle strength (kg)**	32.49 ± 11.30	30.24 ± 11.01	31.95 ± 11.52	30.30 ± 10.83	< 0.001
**BFM (kg)**	19.87 ± 6.64	36.47 ± 6.77	22.80 ± 5.34	36.55 ± 7.39	< 0.001
**FFM (kg)**	46.87 ± 9.20	48.53 ± 9.29	48.23 ± 9.44	50.17 ± 10.22	< 0.001
**BUN (mg/dl)**	13.77 ± 4.10	12.83 ± 3.91	14.00 ± 4.5	13.15 ± 4.12	< 0.001
**Cr (mg/dl)**	1.00 ± 0.21	0.94 ± 0.17	1.02 ± 0.26	0.98 ± 0.19	< 0.001
**eGFR (ml/min per 1.73 m**^**2**^**)**	77.40 ± 14.01	76.44 ± 13.38	74.97 ± 14.13	73.88 ± 13.83	< 0.001
**AST (mg/dl)**	21.32 ± 8.90	20.80 ± 11.10	21.84 ± 8.34	21.58 ± 8.66	0.006
**ALT (mg/dl)**	23.10 ± 13.46	24.91 ± 16.40	27.11 ± 15.50	27.46 ± 15.33	< 0.001
**GGT (mg/dl)**	21.79 ± 17.35	23.92 ± 18.75	28.59 ± 23.16	28.92 ± 20.85	< 0.001
**Energy intake (Kcal/day)**	2684.44 ± 658.51	2672.25 ± 947.10	2589.10 ± 945.34	26.30 ± 955.28	< 0.001
**Carbohydrate (%E)**	61.37 ± 0.10	61.22 ± 0.17	61.34 ± 0.12	61.59 ± 0.17	0.534
**Protein (%E)**	13.67 ± 0.03	13.53 ± 0.06	14.04 ± 0.04	13.92 ± 0.06	< 0.001
**Lipid (%E)**	26.84 ± 0.10	27.42 ± 0.16	26.56 ± 0.12	26.60 ± 0.16	0.002
**Sodium intake (g/day)**	4.81 ± 0.01	4.97 ± 0.04	4.89 ± 0.03	4.93 ± 0.04	0.008

*Abbreviation*: *MHNO* Metabolically healthy non-obese, *MHO* Metabolically healthy obese, *MUNO* Metabolically unhealthy non-obese, *MUO* Metabolically unhealthy obese, *BMI* Body mass index, *PA* Physical activity, *ALT* Alanine aminotransferase, *AST* Aspartate aminotransferase, *CVD* Cardiovascular disease, *BUN* Blood urea nitrogen, *Cr* Creatinine, *GFR* Glomerular filtration rate, *GGT* Gamma-glutamyl transferase, *WHR* Waist hip ratio, *SES* socioeconomic status, *FFM* Fat free mass, *BFM* Body fat mass

^*^P-values were obtained one-way ANOVA and Chi square

Table 3 shows the associations of obesity, MetS, and obesity phenotypes with CKD. After adjusting potential confounders, overweight/obesity was observed to be associated with higher risk of CKD in male subjects (OR = 1.10; 95% CI: 0.78, 1.43); however, this association was not statistically significant. The odds of CKD in male participants with MetS was 88% higher than the odds in non-MetS ones (OR: 1.88; 95% CI: 1.43, 2.45). Accordingly, this association remained significant after adjusting some factors, including age, SES, physical activity, smoking, alcohol use, sodium intake, CVD, GGT, ALT, and AST. The odds of CKD in the MUNO and MUO were 1.70 and 2.20 times higher than those of MHNO phenotype in male cases, respectively. After adjusting the confounders, the odds of CKD in the MUNO and MUO were 1.54 times (95% CI: 1.12, 2.11) and 2.22 times (95% CI: 1.44, 3.41) higher than those of MHNO phenotype in male subjects, respectively. After adjusting the confounders, the odds of CKD in the MUNO and MUO were 1.31 times (95% CI: 1.10, 1.60) and 1.23 times (95% CI: 1.01, 1.54) higher than those of MHNO phenotype in female cases, respectively.

**Table 3:** Association between the obesity, MetS and BMI-MetS categories with the CKD by logistic regression models

	**Number**	**Model I**		**Model II**	
	**Male/ Female**	**OR (95% CI)**		**OR (95% CI)**	
		**Male**	**Female**	**Male**	**Female**
**BMI**					
Normal weight	1694/1156	Ref	-	Ref	-
Overweight &Obese	2947/3867	0.98 (0.74, 1.29)	0.90 (0.60, 1.05)	1.10 (0.78, 1.43)	0.93 (0.77, 1.12)
**MetS**					
No	3363/3108	Ref	-	Ref	-
Yes	1334/1949	**1.88 (1.43, 2.45)**	**1.48 (1.27, 1.73)**	**1.48 (1.10, 2.01)**	-1.14 (0.95, 1.31)
**Obesity phenotypes**					
MHNO	2584/2133	Ref	-	Ref	-
MHO	345/957	0.81 (0.43, 1.52)	**0.71 (0.56, 0.89)**	0.95 (0.49, 1.83)	0.95 (0.74, 1.20)
MUNO	1334/1104	**1.70 (1.26, 2.28)**	**1.51 (1.26, 1.82)**	**1.54 (1.12, 2.11)**	**1.31 (1.10, 1.60)**
MUO	438/867	**2.20 (1.48, 3.27)**	1.16 (0.93, 1.42)	**2.22 (1.44, 3.41)**	**1.23 (1.01, 1.54)**

**Model I:** Unadjusted; **Model II:** Adjusted for age, SES, physical activity, smoking, alcohol use, sodium intake, CVD, GGT, ALT, AST

*MHNO* Metabolically healthy non-obese, *MHO* Metabolically healthy obese, *MUNO* Metabolically unhealthy non-obese, *MUO* Metabolically unhealthy obese

**Fig. 2:**
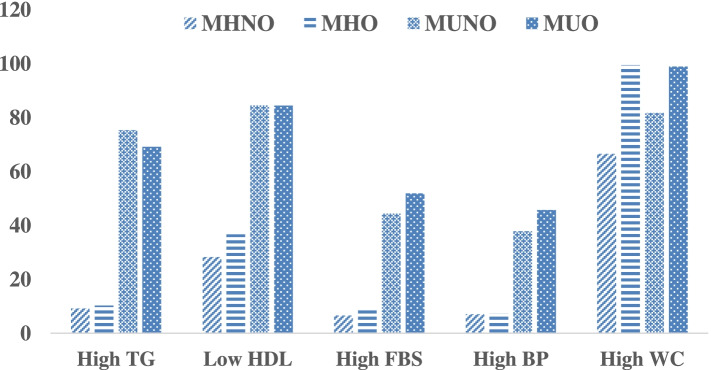
Prevalence of metabolic syndrome components by phenotype obesity

Figure 2 shows the prevalence of MetS components in the four groups defined based on obesity phenotype. Correspondingly, the WC was high in the healthy and unhealthy groups. Moreover, the prevalence rates of all the components of MetS were higher in the metabolically unhealthy groups (MUNO and MUO) compared to the metabolically healthy groups (MHNO and MHO).

## Discussion

In this population-based study on Kurdish adults, the finding showed that MetS was independently associated with higher risk of CKD in male cases. Accordingly, this association remained significant after adjusting potentially confounding variables. In contrast, overweight/obesity had no significant association with higher risk of CKD.

In this regard, previous research has identified overweight/Obesity and MetS as risk factors for CKD, and reported that after controlling the effect of gender, a significant association existed between overweight/Obesity and MetS and an increased risk of CDK [20, 27, 28]. Moreover, in a study by Jay et al., it was shown that the components of the MetS, including disorders of FBS, lipid profile, blood pressure, and central obesity are correlated with low eGFR [27]. However, in this study, we examined the risk of CKD by gender using subgroup analysis, and it was indicated that MetS are associated with odds of CKD, which was not significant in female cases. One of the reasons for the difference between male and female subjects may be the hormones’ effects, which were not measured in this study.

The mechanism of the association between overweight/Obesity and MetS and kidney function is not exactly known yet. Accordingly, one possible mechanism is the number of nephrons, which is determined at birth time, but as weight increases, the GFR of a single nephron must increase as well, in order to keep pace with metabolic needs. According to this hypothesis, individuals born with the lowest number of nephrons will have the highest risk of glomerular hypertrophy if they become obese. This may be due to the reason that obesity requires an additional burden on the nephron, which promotes kidney dysfunction [29, 30]. In addition, MetS, through insulin resistance, leads to the development of a pre-inflammatory condition in the body. As well, the plasma concentration of some pro-inflammatory adipokines increases in MetS patients, while that of other anti-inflammatory adipokines decreases, contributing to the development and progression of kidney diseases [31, 32].

According to the performed analysis, the joint effect of both obesity and MetS as obesity phenotypes was observed, and the odds of CKD in the MUNO and MUO phenotype were found to be significantly higher than those of MHNO phenotype in both male and female cases. Therefore, we conclude that in populations with Kurdish ethnicity, MetS with overweight/Obesity (MetS-obesity) can independently and simultaneously be considered as a risk factor for CKD. In consistent with the findings of our study, a study by Chou et al. showed that Korean female with the MANW phenotype (metabolically abnormal normal weight) are at greater risk for early renal function decline compared to the MHNW (metabolically-healthy normal weight) phenotype [27]. A prospective cohort study on 41,194 Korean adults reported that the risk of CDK was significantly higher in individuals with the MHO and MUNO phenotypes [33]. Moreover, another cohort study of 6,852 Chinese adults with 5-year follow-up reported that both MHO and MUNO phenotypes were associated with the increased risk of CKD [34]. As well, in a prospective study conducted on 3,136 Japanese adults, it was shown that MHO and MUNO phenotypes were not associated with higher risk of CKD [14]. The reasons for this discrepancy may possibly be the low number of new cases, participants underweight in the reference group (MHNO), and the incomplete adjustment of important confounders in the analysis.

The mechanisms of this process have not been identified yet. However, it is assumed that complex pathophysiological factors such as adipocytokines, insulin resistance, renin–angiotensin–aldosterone activation, endothelial dysfunction, and oxidative stress, play a role in this association [35, 36]. The importance of diet and lifestyle (smoking, alcohol use, and physical activity) in kidney function, obesity, and MetS is undeniable, so their effects were adjusted in the analysis.

The most important strengths of the present study are its large sample size and controlling a great number of potential confounders. To the best of our knowledge, this is the first study conducted on Iranian adults with Kurdish ethnicity. Due to the cross-sectional nature of the study, it was not possible to investigate the causal association, which was one of the limitations of the study. Another limitation was that hormones were not measured and their effects on the obesity phenotype and kidneys function were not controlled. However, to prove the generalizability of the results, it is necessary to conduct further research in different regions and with different dietary patterns.

## Conclusion

The present study demonstrated that MetS are independently associated with higher odds of CKD in male cases. In contrast, overweight/obesity was found to be independently, but not significantly, associated with higher risk of CKD. According to the analysis, the joint effect of metabolic obesity phenotype was observed, and the odds of CKD in the MUNO and MUO phenotypes were significantly higher than those of MHNO phenotype in male and female cases. However, we conclude that in populations with Kurdish ethnicity, MetS with overweight/Obesity (MetS-obesity) can independently and simultaneously be considered as a risk factor for CKD. Therefore, lifestyle modification is recommended to control normal weight and healthy metabolism.

## Data Availability

The data sets generated during this study are available from the correspondence author on reasonable request via email.

## Abbreviations

CKD: Chronic kidney disease
MetS: Metabolic syndrome
RaNCD: Ravansar Non-Communicable Diseases
GFR: Glomerular filtration rate
MDRD: Modification of Diet in Renal Disease
MHNO: Metabolically healthy non-overweight/obesity
MUNO: Metabolically unhealthy non-overweight/obesity
MHO: Metabolically healthy overweight/obesity
MUO: Metabolically unhealthy overweight/obesity
CI: Confidence interval
SD: Standard deviation
CKD-EPI: CKD Epidemiology Collaboration
ESRD: Eventually progress to end-stage renal disease
T2DM: Type 2 diabetes mellitus
NCDs: Non-communicable diseases
SES: Socio-economic status
TG: Triglyceride
LDL-C: Low-density lipoprotein Cholesterol
HDL-C: High-density lipoprotein Cholesterol
T-C: Total cholesterol; FBS: Fasting blood sugar
GBD: Global Burden of Disease
CVDs: Cardiovascular disease
IR: Incidence rate
PERSIAN: Prospective Epidemiological Research Studies in Iran
FFQ: Food frequency questionnaire
BP: Blood pressure
DBP: Diastolic Blood Pressure
SBP: Systolic Blood Pressure
PCA: Principal component analysis
GGT: γ-Glutamyltransferase
ALT: Alanine aminotransferase
AST: Aspartate aminotransferase
BMI: Body mass index
BFM: Body fat mass
FFM: Fat free mass
WHR: Waist-to-hip ratio

## Footnotes

1 Prospective Epidemiological Research Studies in Iran.
